# T cells responses after vaccination: a regulatory perspective

**DOI:** 10.3389/fimmu.2025.1584738

**Published:** 2025-06-12

**Authors:** Alessandra Buoninfante, Marco Cavaleri

**Affiliations:** Public Health Threats Department, European Medicines Agency, Amsterdam, Netherlands

**Keywords:** vaccines, T cells, assays, regulatory approval, decision making, pathogens, cell-mediated immunity

## Abstract

Vaccines are complex biological medicinal products developed with the aim to generate protective immunity against specific infectious diseases in a particular target population. Regulatory authorities, who have the role of approving vaccines, ensure that these meet the agreed criteria for quality, safety and efficacy, and assess their benefit and risk profile before and after a marketing authorization is granted. In the European Union/European Economic Area, the vast majority of the vaccines currently available has been approved on the basis of clinical efficacy or immunogenicity data relying on humoral immune responses. Per contrary, there are no vaccines approved based on immunogenicity endpoints exclusively focused on cell mediated immunity, despite the known relevance of T cells immunity for protection against a variety of infectious diseases. We here review a few relevant cases of vaccines targeting infectious diseases for which data on cell mediated immunity have been considered in the context of regulatory filing, and provide our perspective on the way forward.

## 1 Introduction

Vaccines against infectious diseases aim to protect us from pathogens by activating antigen specific B and T cell responses. B cells produce antibodies, which can neutralize the pathogen by direct binding or by blocking certain functions of the pathogen and can exert immunological effector functions.

T cells recognize foreign antigens as short peptides presented on the cell surface in complex with human leukocyte antigen (HLA) class I or class II molecules, which confers them high specificity. Upon recognition of their cognate peptide presented by HLA molecules, memory T cells can rapidly elaborate effector functions to suppress replication, limit infection, and prevent spreading of pathogens within the host (1). Therefore, when designing vaccines against infectious diseases ideally both B and T cell responses should be targeted.

The European Medicines Agency (EMA) is the regulatory body responsible for the evaluation of centralized marketing authorization applications of medicinal products in the European Union/European Economic Area. This includes the evaluation of all new vaccines, and with its committees, EMA assesses their quality, safety and efficacy. The EMA assesses the scientific data submitted by developers on a specific products and grant its approval only if the benefit/risk ratio is considered positive for a defined indication of use. For vaccine approval, if an immune correlate of protection (CoP) defined as a type and amount of immunological response that correlates with vaccine-induced protection against an infectious disease (2) is available, it is deemed suitable to infer protection and demonstrate clinical benefit. Alternatively, if there is no established CoP and field efficacy studies are problematic, another approach to support approval of new vaccines is to use immune markers that are likely to predict protection even if the correlate of protection is not fully established.

All currently licensed vaccines generate humoral immune responses and work by either preventing infection or disease. Several approved vaccines have defined levels of serum antibodies that can be easily quantified and serve as correlates or surrogates of protective immunity. For these vaccines analysis of serum antibody titers provides clear and reliable information on vaccine efficacy and protection from disease. Emblematic representative cases are the correlates of protection of Hepatitis A, Hepatitis B, Hemophilus influenzae, where defined levels of antigen specific antibody levels measured by ELISA have been linked to protective efficacy and are used to support the authorization of a given product (3). Assays such as ELISA or neutralization assays which measure antibody titers have been standardized and used for licensure over the last decades. Differently than antibody responses, analysis of antigen specific CD4+ and CD8+ T cell responses and defining their contribution to vaccine-mediated immunity have been and remains a challenge. This is primarily due to the existence of many different sub- types of T cells with not completely defined functions and to their different tissue distribution. Additional challenges are encountered when trying to establish reproducible and reliable quantitative measurements.

In this article we reflect on the importance of eliciting T cells immunity with vaccination and provide a regulatory perspective on current state of play when it comes to documentation of T cells responses for regulatory submissions and decisions for vaccines, using specific case-examples.

## 2 T cell immunological assays and current challenges

Identifying cell-mediated responses that correlate with efficacy is still challenging, and in fact no vaccine so far has been approved based only on T cell responses.

The phenotype of T cells can be assessed on a single-cell level by exploiting specific markers which define their functionality. Some of the assays identify T-cells on the basis of surface markers (immunophenotyping, tetramers) or cytokine release (functionality-based cytokine measurements) or T-cell effector function assays. Enzyme-linked immune absorbent spot (ELISpot) is a method for measuring relevant parameters of T cell activation and it is frequently used for quantifying antigen-specific cellular responses after vaccination in clinical trials. It is high throughput, robust and relatively economical, which makes it particularly suitable for measuring responses in clinical samples. Differently, intracellular cytokine staining (ICS) and other analyses by flow cytometry provide an advantage over ELISpot as they allow both multiparameter cytokine analysis and phenotyping from more cells, but these assays are more laborious and more difficult to standardize.

Whilst both ELISpot and ICS have a clear role in quantifying T-cell responses, there are a range of other tools which can be applied to assess T-cell responses to vaccination, both pre-clinically and in clinical trials. Very popular are the platforms which measure soluble analytes like cytokines in biological matrixes, essentially providing read-outs like a multiplex ELISA.

Maintaining the integrity of the clinical sample is crucial when performing cell-mediated immune analysis. Indeed, significant logistical challenges are encountered when trying to collect samples for performing such assays in clinical trials and reliably determine quality and quantity of T cells responses. Cellular assays have also significant costs, lower throughput, and require larger sample volumes, which becomes particularly limiting when having to collect e.g. pediatric clinical samples.

Inter- and intra-laboratory variability should be minimized to help data interpretation, for example sample handling or technology platforms should be optimized to allow reproducibility between tests and decrease the bias accumulated during the manipulation workflow. In this context, particularly relevant are the development of standard operating procedures, also for data acquisition and training of personnel. Additional unresolved issues on assays to measure cell-mediate immunity in the context of regulatory submissions are the identification of adequate quality controls, including in settings like COVID-19 or influenza where the viruses change frequently.

A major biological complication in trying to standardize cell-mediated assays is driven by the extensive diversity within the human population in terms of MHC genes, which impact the breadth and diversity of T cell repertoire responses. The difficulty in measuring T cell responses is also aggravated by the compartmentalization of T cell subpopulations and their migration across locations. Indeed, the accessibility of T cells in immunologically relevant locations of human body can be complicated, i.e. for mucosal sites. Additionally, T cell responses are influenced by the timing when an assay is performed. It is known that there are huge differences between the frequencies of immune populations according to the tissues source, the cryopreservation method used or the human physiological rhythm.

All these aspects lead to the difficulty of generating solid data on correlates of protection based on cell-mediated immunity (CMI) and to achieve a sufficient level of standardization and validation of the assays. Method validation aims to demonstrate that it is suitable for its intended purpose through the provision of objective evidence that specified requirements have been fulfilled (4). The main objective of method validation is to demonstrate the reliability of a particular method for the determination of an analyte concentration in a specific biological matrix (5). In this context, it is always necessary for the primary endpoint assay of a pivotal clinical trial to be validated, as defined by the EMA guidelines on clinical evaluation of vaccines (2), but the choice of the validation approach can be per se a crucial decision to make.

For the design and evaluation of next generation vaccines aiming at the induction of cellular immune responses the development of well standardized high throughput assays is paramount.

## 3 Regulatory cases

According to the EMA (2) and WHO (6) guideline for the clinical evaluation of vaccines targeting infectious diseases, vaccine candidates have to be tested for cellular immune responses as part of their development program in clinical trials. Characterization of the immune response in biological matrices could comprehend the assessment of the cell-mediated immunity, for instance by quantifying vaccine antigen/s specific T-cells. Also, primary and secondary endpoints reported from comparative immunogenicity trials may include pre- and post-vaccination numbers or percentages of subjects with antigen-specific T-cells (including CD4+ and CD8+ T-cells).

Because protective efficacy of T cell responses is hard to establish and to statistically infer, T cell immunity is always investigated secondarily or exploratively in clinical studies, and therefore so far it has not been used as pivotal data for regulatory decision making.

The importance of T cells for supporting regulatory approval of a vaccine candidate has been long debated by experts in the field of infectious diseases (7). Although cell mediated immunity alone has not been used for this purpose, on some occasions T cells data have provided important information that contributed to the development of vaccines or to the understanding of its protective mechanism.

We here review a few recent cases within the regulatory framework of vaccine approval with a focus on viral vaccines.

### 3.1 COVID-19

At the start of COVID-19 pandemic, for the approval of COVID-19 vaccines targeting the parental SARS-CoV-2 strain a large-scale phase 3 efficacy trial was required for the marketing authorization application. For this pivotal study the primary endpoint was laboratory-confirmed COVID-19 disease of any severity and the primary efficacy analysis was restricted to subjects seronegative for SARS-CoV-2 at baseline. The first approved COVID-19 vaccines were the mRNA-based vaccine, Comirnaty (8) and Spikevax (9), for which indeed a phase 3 vaccine efficacy trial served as basis for approval. Immunogenicity analysis based on functional antibody responses were secondary objectives, while CMI was measured only exploratorily. For both vaccines, the applicants submitted data generated in a subset of participants from Phase 1 trials which showed a clear Th1-polarised response upon vaccination, allowing exclusion of the theoretical risk of vaccine dependent enhancement of disease, which has been linked to Th2 cells (10). Despite extensive CMI characterization was not done at the time of approval, regulators considered these data not critical for the benefit-risk assessment of the vaccines, also because no concerning effects emerged from preclinical tests and the efficacy trials.

Later on, the EMA recommended approval of new vaccines on the basis of clinical immunobridging studies showing noninferiority or superiority of the amount of neutralizing antibodies elicited by the new vaccine compared to an approved vaccine that demonstrated efficacy (11).

Indeed, it has been shown that neutralizing antibodies are highly predictive of immune protection from symptomatic SARS-CoV-2 infection (12), but particularly with the emergence of SARS-COV-2 variants it became clear that neutralizing antibodies are not the only players in protective immunity (13), and that other components of the immune response also contribute to protection especially from severe disease (14). Even initial data from efficacy studies showed protection after first dose, when the amount of antibodies is very low, hinting to other contributing mechanisms.

The role of T cells in protective immunity to SARS-CoV-2 has been demonstrated by evidences in cancer patients with B cell deficiencies affected by COVID-19, where higher CD8+ T cell responses correlated with milder disease (14). Additionally, robust protection against severe disease in the absence of high neutralizing antibody titers (15) (16) again suggested that T cell responses likely contribute to it.

In the context of COVID-19 booster doses, differently than B cell immunity which require constant vaccine adaptation as neutralizing antibodies do not efficiently neutralize antigenically distinct variants, T cell responses appeared to be conserved across the different SARS-COV-2 variants reiterating their contribution for protection against severe disease.

Importantly, the durability and reactivity of CD8+ T cells against different SARS-COV-2 variants that escape neutralizing antibodies (17) (18) (19) (20) suggested their relevance for preventing severe disease.

Controlled human infection models (CHIM) were particularly helpful to understand the role of CMI in the context of prevention of infection. In a COVID-19 CHIM trial (21), intranasal inoculation of SARS-CoV-2 virus in 34 seronegative 18–30-year-old volunteers was able to infect 53% of them. A positive correlation was seen between viral load and the timing and size of IFN-y responses, suggesting that viral load drives T cell responses. Furthermore, a negative correlation was found between the size of the total activated T cell response with the viral load peak (21), underpinning the role of CMI.

### 3.2 Influenza

The immunogenicity of influenza vaccines is mostly measured by the hemagglutination inhibition assay that detect antibody directed against the hemagglutinin (HA) antigen.

While no correlate of protection is considered formally established, the use of HAI titres and the threshold of antibodies in sera with a dilution 1:40 has been used since many years as a surrogate endpoint for traditional influenza vaccines, i.e. vaccines based on HA antigen manufactured as purified proteins or split virus. The shortcomings with such threshold and the use of HAI assays, for example in the context of avian influenza, have prompted the investigation of neutralization assays as a possible alternative.

Measurement of CMI is never considered mandatory to support regulatory decision making, but it is of value particularly in the elderly due to immunosenescence, as it is reflected in the current EMA guidelines (2). However, in the majority of authorized seasonal influenza vaccines, CMI has not been measured and not considered instrumental for the final decision on the risk/benefit.

CMI data were provided as part of the application package of the zoonotic influenza inactivated and adjuvanted H5N1 (A/turkey/Turkey/1/2005 strain) vaccine Aflunov (22). For this vaccine, in a phase 2 study conducted in healthy adults (23) (22), which was submitted to the regulators, CMI of two different doses of Aflunov followed by a booster dose was compared to the non-adjuvanted vaccine. Only the adjuvanted vaccine was able to induce an increase in the frequency of antigen specific CD4 T-cells, which also displayed an effector/memory Th1 phenotype. Inactivated unadjuvanted vaccines were not able to elicit CMI. For the adjuvanted formulation, although results looked promising and the data generated were deemed important by regulators to further characterize vaccine induced immune response in humans, it was considered that the implications of CMI read-outs could not be reliably interpreted at the time of approval due to lack of an established correlate of protection. Of note, CMI data were intended to be the primary objectives of another clinical trial, but they were lacking at the time of opinion and were demanded by regulators as post-approval commitment.

Also for the split virion, inactivated, adjuvanted pandemic influenza vaccine (H5N1) Pandemrix (24) CMI responses and T cells cross-reactivity against several heterologous strains were measured. Frequencies of influenza-specific CD4 T-cells were higher in adjuvanted compared to the non-adjuvanted groups and remained higher than pre-vaccination status up to 180 days post vaccination, while no significant effect of vaccination was observed on the influenza-specific CD8 T-cells. Additionally, the adjuvanted vaccine was able to elicit a significant increase in the response against HA peptides of A/Vietnam, A/Indonesia and A/Anhui strain compared to the unadjuvanted vaccine. Of note, case reports from some EU Nordic countries described narcolepsy in children following vaccination with Pandemrix (25,26). Narcolepsy is a chronic sleep disorder with a suspected autoimmune etiology, and when associated with hypocretin deficiency is linked to a genetic predisposition (HLA-DQB1^*^06:02). To evaluate the association between narcolepsy and Pandemrix, several epidemiological and mechanistic investigations were conducted post approval. Three hypotheses were explored: molecular mimicry, bystander activation and inflammation/damage to the hypothalamus (27). The majority of data generated so far considered most likely the CD4 T cell cross-reactivity between specific epitopes in hemagglutinin HA (HA275-287) and hypocretin sequence (HCRT56-68, HCRT87-99).

Besides protective antibodies, there is the notion that influenza-specific cytotoxic T cells also have an important protective role in mitigating the severity of influenza disease (28). Protection conferred by inactivated influenza vaccines, which is based on antibodies directed against the surface proteins of the influenza viruses, i.e hemagglutinin and neuraminidase, can theoretically be improved by inducing T-cell responses to conserved internal influenza antigens. In humans, a few challenge studies and large observational studies support the role of T cell responses in reducing symptomatic influenza-associated disease but not for prevention of infection (29). The magnitude of T cells measured in the peripheral blood induced by vaccination has not yet proven to be a correlate of immunity. A recent interesting study of influenza controlled human infection challenge investigated if susceptible individuals receiving a vaccine boosting T-cell responses would exhibit lower viral load and decreased symptoms compared to placebo (30). In this study, an MVA based influenza vaccine induced the highest T-cell responses against non-surface proteins, matrix protein 1 and nucleoprotein, but there was no clear evidence that vaccine-induced responses to these proteins alone were associated with protection.

Various studies have shown protection after live attenuated influenza vaccine (LAIV) also during influenza seasons with strain mismatch, indicating that these vaccines are able to induce a broader cross-protective immunity. Because LAIVs replicate in host cells, they are able to induce CD4+ and CD8+ T-cell immunity. Correlates of protection for LAIV are yet to be established, but attempts have been made. Notably, recent studies have found that multifunctional CD4+ T cells were elicited by H5N1 vaccination suggesting they may be used as a correlate (31,23).

It is expected that a robust T-cell response induced by vaccination would protect against complications or severe disease and therefore be particularly relevant for older adults, i.e. above 65 years of age, due to immunosenescence. Indeed, a study in elderly has shown that humoral immune responses were comparable between subjects with influenza illness and the ones with no signs of disease (32). As measured by validated Granzyme B assays *in vitro*, higher levels of granzyme B, which is major constituent of cytotoxic T cells and natural killer cell granules, in elderly subjects were associated with protection from influenza illness up to 10 weeks post-vaccination.

Several strategies have been applied to improve influenza vaccines for the elderly, including the increase of the vaccine antigen per dose, the administration of the vaccine by intradermal versus intramuscular route and addition of adjuvants (33). Also, preliminary data have shown that conserved T cell epitopes expressed by diverse influenza strains can induce broadly protective influenza immunity, providing the rationale for further development of broad spectrum influenza vaccines targeting T cells epitopes (34). Future investigations aiming at evaluating if a greater magnitude of T-cell response or localization of T cells in the lungs can be elicited by using intranasal or aerosol delivery, and whether this is associated with increased clinical protection, would be warranted.

### 3.3 Ebola

Ebola virus disease is rare but severe and often fatal. The largest outbreak so far occurred in West Africa in 2014–2016 with more than 11,000 deaths. A lot of what we know about immune-biomarkers and the role of T cells for Ebola virus disease comes from animal data.

Experiments in monkeys conducted with the investigational Ebola chimpanzee adenovirus vectored (Ad5) vaccine suggested that cellular immunity is required for virus clearance (35). In a study conducted in macaques, passive transfer of polyclonal antibodies from Ebola Ad5 vaccinated macaques to naïve macaques (n= 4) failed to confer protection against disease in 3 of 4 animals, suggesting a limited protective effect of humoral immunity. Per contrary, depletion of CD8+ T cells abrogated protection in most of the animals suggesting that CD8 + T cells are required for complete protection induced by Ad5-Ebola vaccination (36).

Another investigational vaccine, a replication-defective recombinant chimpanzee adenovirus type 3–vectored ebolavirus vaccine (cAd3-EBO), encoding the glycoprotein from Zaire and Sudan species, that showed protection in a nonhuman primate model, was tested in phase 1, dose-escalation, open-label trial (37) where glycoprotein-specific antibodies and T cells were measured at 8 weeks and 48 weeks post vaccination. Most of the memory CD4 and CD8 glycoprotein-specific T-cell responses were polyfunctional and a high proportions of CD8 cells produced both IFN-y and TNF, which had been linked to protection in nonhuman primates (38). Polyfunctional T cells are able to carry out multiple functions, such as the secretion of different cytokines, chemokines, or cytotoxic granules simultaneously at the single cell level. It has been suggested that the appearance of polyfunctional T cells is a sensitive immune correlate for immunological disease control. The benefit of poly-functional T cells in protection against pathogens has been attributed by their ability to express higher levels of IFNγ on a per-cell basis than other populations; enhanced cytotoxicity due to secretion of both IFNγ and TNFα and high IL-2-mediated proliferative capacity.

Interestingly and differently from studies using adenovirus as vector, in a study using the cynomolgus macaques infection models, it was shown that antibodies to Ebola Zaire induced by the vesicular stomatitis virus (VSV) vaccine platform were able to protect animals against lethal challenge, while CD8 and CD4 T cells had a marginal role to protection, as shown in CD20+, CD4+ or CD8+ depletion experiments, that however used different depletion conditions than the Adenovector-based vaccines studies (39). Data generated by researchers post approval have shown that there might be a role for CD8+ T cells mediated protection, not identified previously (40).

Zabdeno (41), and Mvabea (42), an Ebola Ad26 vectored recombinant vaccine with heterologous boost by an MVA based filovirus vaccine was approved by the EMA on the basis of a bridging strategy. Given the unfeasibility of conducting a human efficacy trial, clinical immunogenicity results were bridged to data obtained in non-human primates. To translate human immunogenicity data into likelihood of protection, a logistic regression model was built based on immunogenicity and efficacy data obtained in the non-human primates Ebola Zaire virus lethal challenge model, which closely resembled the human Ebola Virus disease, and human immunogenicity data.

To build the logistic curve, binding antibodies in animal sera and human samples from the immunogenicity trials as measured 21 days post second dose were used.

A few challenge studies were performed in cynomolgus monkeys, with the highest protective efficacy obtained using a dosing interval of 56 days which showed that survival was 100%, when challenged around the peak in antibody levels (approximately 4 weeks after the second dose). A strong correlation between binding and neutralizing antibodies was recorded, but glycoprotein-binding antibodies was chosen as the parameter for the immunobridging strategy, considering the robustness of the glycoprotein-binding antibody assay compared to the neutralizing antibody assay.

T cell responses had a limited contribution to the discriminatory capacity of the binding antibody levels in the prediction model, therefore it was considered that for the assessment of the benefit/risk of the vaccine CMI data were not instrumental. For this reason, the immunobinding studies supporting the approval were conducted based on the amount of binding antibodies in humans and not on CMI data, despite their mechanistic role in protection.

### 3.4 Shingles

Shingles is a debilitating disease characterized by a vesicular rash, with the most common complication being postherpetic neuralgia. The currently approved vaccines consist of live varicella virus or adjuvanted viral glycoprotein E (gE), both eliciting antibodies and cellular responses. The disease risk increases in people 50 years and older due to immunosenescence or in immunocompromised people. To date, a statistical correlation with antibodies to viral glycoproteins was shown.

Initial zoster vaccine development focused on producing a live, attenuated varicella-zoster vaccine that could elicit CMI responses to a broad spectrum of viral antigens. For ZOSTAVAX^®^, a live attenuated varicella-zoster virus (VZV) vaccine, efficacy and safety were studied in one pivotal randomized, double-blinded, placebo-controlled, multicenter study. A sub-study was conducted to further evaluate cell mediated immunity and potential CMI correlates of protection against herpes zoster and postherpetic neuralgia (43). As part of the dossier submission, in terms of IFN-y ELISpot counts, the zoster vaccine elicited significantly higher specific immune responses compared to placebo, which persisted above baseline up to 36 months. In order to evaluate whether the vaccine VZV-specific immune responses correlated with protection against herpes zoster, data generated by VZV IFN-γ ELISpot assay and glycoprotein ELISA were analyzed according to the herpes zoster status. This analysis was considered inconclusive because the sub-study represented only 3.6% of the total study population, and too few participants developed herpes zoster during the study. Also, correlation between the immune responses and protection against herpes zoster were observed as measured by a glycoprotein ELISA, while, the results of ELISpot test had a far less clear correlation to the protection.

While studies directly linked to the marketing authorization showed that IFNγ responses increase upon vaccination, the quality of the T cell response has been elucidated only after approval. By using polychromatic flow cytometry, the breadth, magnitude, and quality of CD4^+^ and CD8^+^ T cell responses induced 3–4 weeks after ZOSTAVAX vaccination of healthy adults with a history of chicken pox was characterized (44). The investigators found that the highest frequencies of VZV-specific CD4^+^ T cells were poly-functional CD154^+^IFNγ^+^IL-2^+^TNFα^+^ cells, which were boosted upon vaccination. These results were achieved only by using overnight stimulation of PBMC with live VZV and the activation of PBMC for several days, instead of standard protocols, indicating that assay adjustment is key.

Developers of Shingrix (45), an adjuvanted vaccine against shingles consisting of the glycoprotein E antigen and the AS01B, evaluated and submitted CMI responses post vaccination as these are considered key in the prevention of VZV reactivation. Since it is considered that CMI responses are essential for protection against the development of herpes zoster, regulators requested a comprehensive overview of T cells analyses. Indeed, CMI responses were evaluated in several Phase 1, 2 and 3 trials to characterise the immunogenicity of the vaccine and to establish the optimal vaccine dose and formulation. Shingrix elicited strong and persistent CD4+ T cell responses, which possibly contributed to the efficacy of the vaccine. Importantly, it was attempted to define a CoP based on a pre-specified endpoint for the pivotal phase III efficacy trials, where they conducted an analysis of herpes zoster breakthrough cases. Although it is generally assumed that the immune mechanism of protection against the virus reactivation is mainly cell-mediated, investigators decided to study a potential correlation between efficacy and the anti-gE antibody response 1 month post-vaccination because of practical reasons linked to having an established and reliable ELISA assay. Despite the analyses conducted at individual and population level suggested that anti-gE antibodies are a valid immune marker to support regulatory decision making in healthy subjects, the actual model linking anti-gE to probability of protection remained not completely understood and therefore, at the time of approval the CoP could not be considered established. For this reason, it was also agreed that immunobridging based on anti-gE antibodies to extend the use of the vaccine to other subgroups or circumstances will have to be defined on a case-by-case basis.

### 3.5 Dengue

The immune response to dengue infection is very complex and involves both the humoral and cellular branch of the immune system, but the exact mechanisms that underlie protective immunity are not yet fully comprehended.

For the recently authorized live, attenuated, dengue tetravalent vaccine (46), Dengue Tetravalent Vaccine Takeda, a possible correlate of protection was explored on the basis of neutralising antibody titers to predict vaccine efficacy from dengue infection. In a descriptive analysis, GMTs were compared between subjects who had virologically-confirmed dengue fever until 18 months post second dose of the vaccine and those who did not have fever, referred to as controls. It emerged a potential association between the neutralising antibody titre and preventing virologically-confirmed dengue fever. This was particularly evident in baseline seropositive subjects. Unfortunately, from the study it was not possible to identify a specific cut-off titre as predictive of clinical protection due to the considerable overlap in antibody titres between cases and controls, particularly in the baseline seronegative study group.

Furthermore, while in the pivotal study to support vaccine approval, it was seen a waning of vaccine efficacy from first to third year after immunization, a similar decline was not recorded in terms of GMT titres and seroprotection rates, which indicates that other elements of the immune system contribute to preserve long term vaccine efficacy.

To study vaccine induced cell mediated immunity, samples have been collected from participants in clinical studies and submitted to regulatory authorities, which showed that the vaccine elicits cellular immune responses in adults, adolescents and children. IFN-γ ELISpot assays with peptide pools spanning the DENV proteome demonstrate that cellular immune responses were elicited to all vaccine components. ICS identified a higher magnitude of CD8+ T cells compared to CD4+ T cells in addition to multi-functional CD4+ and CD8+ responses, with IFN-γ+/TNF-α+ or IFN-γ+ CD8+ cells being the most frequent phenotype. As cell mediated immunity to the vaccine was only studied in exploratory manner, these data did not inform any regulatory decision.

## 4 A regulatory perspective and outlook to the future

The examples provided shows on one hand that advancement in our ability to understand the role of CMI has taken place, but on the another hand also highlighted that it is not yet possible to use CMI biomarkers as correlates of protection or as quantitative immune markers for immunobridging. Although T cell data has at times been sent by developers to regulators in their submissions for approval (see above and **Table 1**), these data have not been amenable to be used for regulatory decisions.

**Table 1:** Schematic overview of CMI data as part of initial regulatory submission per vaccine.

Disease	Product name	Regulatory submission
COVID-19	Comirnaty Spikevax	- CMI as exploratory analyses in Phase 1 studies ((enzyme-linked immunospot assay (ELISpot) and intracellular cytokine staining) - CMI as exploratory analyses in Phase 1 studies (intracellular cytokine staining)
Influenza	Aflunov Pandemrix	- CMI data generated in Phase 1 and 2 clinical studies (intracellular cytokine staining) - CMI as supportive evidence in main immunogenicity studies (intracellular cytokine staining)
Ebola	Zabdeno	- CMI as exploratory analyses in Phase 1 and Phase 1-2 studies(ELISpot and intracellular cytokine staining)
Shingles	Zostavax Shingrix	- CMI data generated in Phase 2b and in a substudy of a Phase 3 trial (ELISpot and RT-PCR) - CMI data generated in Phase 3 trial and in Phase 1-2 and Phase 2 supportive immunogenicity studies (intracellular cytokine staining)
Dengue	Dengue Tetravalent Vaccine Takeda	- CMI data generated as primary or secondary trial objectives in Phase 2 studies (ELISpot and intracellular cytokine staining)

As we know for many vaccines, the induced T cells responses play an important role in protection from disease, we need to reflect on the actions that are required to collect and use CMI data in the context of regulatory activities.

Despite the improvements in the assays used, it is widely acknowledged among experts that significant variability especially across laboratories still exists. This aspect together with the difficulty in collecting large number of samples at the most relevant biological time point and from the most pertinent tissue in vaccinated individuals further complicate the interpretation of data and their ability to provide the needed strength to assess potential correlation. Many attempts, including studies based on ICS approaches led to interesting data from a qualitative perspective, i.e. spectrum of polyfunctional T cells for Ebola (37, 47) but could not be used to derive a quantitative estimates or even putative thresholds.

As a first step, attempts to measure T cells responses should be systematically considered for any new vaccine in development, especially when it is known that CMI responses are likely to play a role in protection. Datasets should be sufficiently large to allow testing for correlation with protection (48), with the aim to show that CMI responses are responsible for and statistically interrelated with protection from a specific infection or disease.

To reduce the numerous technical challenges, high-throughput approaches for sample collection, sample storage, reagents preparation and data analyses, could aid in developing more reliable assays. In this context, development of standard protocols for each assay, ensuring reference materials and quality-controlled reagents are key points for assay harmonization. Recent advancements in assay robotics have already led to promising results and could further be employed to increase assay reliability. Furthermore, establishing reference laboratories which ensure high quality of infrastructure and operations should be also considered.

To generate data which can support regulatory decisions it is paramount to conduct well designed and sufficiently powered clinical studies. Converging experts group could be a plausible way forward to reach alignment, including seeking input from regulators. Indeed, *ad hoc* workshops could be a valuable tool to bring together regulators, scientific experts and developers to discuss and agree on possible solutions. The EMA Emergency Task Force (49) has been and intends to be the place where these issues can be discussed and tackled. Developers and academics are invited to discuss with the ETF cross-cutting science on CMI and to build public-private partnership to advance the field. In addition, to support the qualification of innovative development methods for a specific use in the context of research and development of medicinal products the EMA offers scientific advice procedures (50).

In terms of actually showing correlation to protection, it is acknowledged that defining the individual contribution to clinical protection of B and T cells is quite complex due to the redundancy of the immune system. Mechanistic studies in animal challenge models where individual cell populations were depleted to pinpoint their contribution to protective efficacy, as seen for the development of Ebola vaccines, are one way to help understanding the individual contribution of B and T cells in conferring protection from disease. However, the results of depletion studies may sometimes appear contradictory (35, 39). Clinical studies enrolling immunocompromised subjects lacking defined immune-components could provide mechanistic and biological insights on how the immune system operates and help the field to progress in understanding the impact of specific immune responses on protection. Indeed, researchers found that in patients with hematologic cancer and impaired antibody responses hospitalized for COVID-19, CD8 T cell counts were associated with patients survival. Subjects with the lowest SARS-CoV-2 specific T cells, had higher disease severity and mortality, regardless of the magnitude of B cell response, while patients with more robust T cell responses had less severe disease and lower mortality (14). In another study (51) it was shown that B-cell depletion after rituximab treatment in patients with immune-mediated glomerulonephritis and vasculitis was significantly associated with failure to seroconvert, although most of these patients maintained the capacity to generate T-cell responses to SARS-CoV-2 vaccination. While these studies would be enlightening the relevance of T cells responses for protective immunity, it may still be difficult to derive from their results clear-cut correlates of protections that could be used in more generalized populations and for regulatory purposes.

With the fast advancement of technology, it should be possible to develop new sophisticated methods that reliably define quantitatively and qualitatively T cell responses and use them to infer vaccine efficacy. For example, an innovative method to measuring adaptive immunity consists in the use of immune organoids from tonsils, which has shown promising results in the influenza field (52). New methods are needed to expand current capabilities, by utilizing system’s biology approaches and artificial intelligence.

Systems vaccinology applies ‘omics’ technologies to study immunological responses to vaccination (53), with the aim to characterize the interactions between individual components of the immune system to understand and predict behavior of the system as whole. This kind of studies allows in-depth analysis of T-cell populations that are not possible through individual cytokine analyses alone and may provide complementary useful information to more traditional investigations. In this context, some studies have reported promising examples of transcriptomic signatures in vaccine studies which predicted vaccine efficacy (54).

Single-cell T cell and B cell antigen receptor-sequencing data analysis can potentially perform in-depth assessments of adaptive immune cells and inform our understanding of immune cell development (55). The recent COVID-19 pandemic offered some interesting ideas for further reflection. Indeed, some researchers used sequencing technologies to perform a longitudinal analysis of circulating human leukocytes collected before and after COVID-19 vaccination (56). By using cross-modality integration tools, investigators defined their transcriptome, accessible chromatin landscape and immunophenotype, and identified unique biomarkers. Also, by using scRNA-sequencing in COVID-19 patients, they showed that CD8+ T cell populations had relative frequency and differentiation outcomes predictive of subsequent clinical outcomes. This demonstrated the potential for monitoring antigen-specific T cells to represent features of human immune responses more broadly and thereof, inform understanding of disease and response to vaccination.

Future efforts should be directed towards new experimental and computational techniques that permit the integration of sequence, phenotypic, and functional information. The size and complexity of this task imply the titanic effort of combining the latest immunological understandings of cellular immunity with developments in the field of data science and artificial intelligence. Ultimately, to achieve regulatory outcomes it is essential that scientific findings are validated in clinical studies, where reliable clinical samples and robust assays are employed.

## References

[1] Wherry EJ Barouch DH T cell immunity to COVID-19 vaccines. Science. (2022) 377:821–2. doi: 10.1126/science.add2897 35981045

[2] EMA . Available online at: https://www.ema.europa.eu/en/clinical-evaluation-new-vaccines-scientific-guideline. (Accessed February 18, 2025).

[3] Walter A Orenstein PAO Edwards KM Plotkin, 8th edition. Philadelphia, PA: Elsevier (2023).

[4] EMA . ICH M10 on bioanalytical method validation - Scientific guideline.

[5] EMA . (2022). Available online at: https://www.ema.europa.eu/en/documents/scientific-guideline/ich-guideline-m10-bioanalytical-method-validation-step-5_en.pdf. (Accessed February 18, 2025).

[6] WHO . Available online at: https://www.who.int/publications/m/item/clinical-evaluation-of-vaccines-annex-9-trs-no-1004. (Accessed February 18, 2025).

[7] Marchant A Van Damme P Plotkin S Neels P Cassetti MC Cramer J . Enabling the evaluation of COVID-19 vaccines with correlates of protection. Biologicals. (2024) 85:101723. doi: 10.1016/j.biologicals.2023.101723 37976940

[8] EMA . Available online at: https://www.ema.europa.eu/en/medicines/human/EPAR/comirnaty. (Accessed February 18, 2025).

[9] EMA . Available online at: https://www.ema.europa.eu/en/medicines/human/EPAR/spikevax. (Accessed February 18, 2025).

[10] Gartlan C Tipton T Salguero FJ Sattentau Q Gorringe A Carroll MW . Vaccine-associated enhanced disease and pathogenic human coronaviruses. Front Immunol. (2022) 13:882972. doi: 10.3389/fimmu.2022.882972 35444667 PMC9014240

[11] ICMRA . ICMRA COVID-19 Omicron variant workshop. (2022).

[12] Khoury DS Cromer D Reynaldi A Schlub TE Wheatley AK Juno JA . Neutralizing antibody levels are highly predictive of immune protection from symptomatic SARS-CoV-2 infection. Nat Med. (2021) 27:1205–11. doi: 10.1038/s41591-021-01377-8 34002089

[13] Peng Y Mentzer AJ Liu G Yao X Yin Z Dong D . Broad and strong memory CD4(+) and CD8(+) T cells induced by SARS-CoV-2 in UK convalescent individuals following COVID-19. Nat Immunol. (2020) 21:1336–45. doi: 10.1038/s41590-020-0782-6 PMC7611020 32887977

[14] Bange EM Han NA Wileyto P Kim JY Gouma S Robinson J . CD8(+) T cells contribute to survival in patients with COVID-19 and hematologic cancer. Nat Med. (2021) 27:1280–9. doi: 10.1038/s41591-021-01386-7 PMC8291091 34017137

[15] Cele S Jackson L Khoury DS Khan K Moyo-Gwete T Tegally H . Omicron extensively but incompletely escapes Pfizer BNT162b2 neutralization. Nature. (2022) 602:654–6. doi: 10.1038/s41586-021-04387-1 PMC8866126 35016196

[16] Gray G Collie S Goga A Garrett N Champion J Seocharan I . Effectiveness of ad26.COV2.S and BNT162b2 vaccines against omicron variant in South Africa. N Engl J Med. (2022) 386:2243–5. doi: 10.1056/NEJMc2202061 PMC9093716 35507482

[17] Collier AY Yu J McMahan K Liu J Chandrashekar A Maron JS . Differential kinetics of immune responses elicited by covid-19 vaccines. N Engl J Med. (2021) 385:2010–2. doi: 10.1056/NEJMc2115596 PMC8531985 34648703

[18] Goel RR Painter MM Apostolidis SA Mathew D Meng W Rosenfeld AM . mRNA vaccines induce durable immune memory to SARS-CoV-2 and variants of concern. Science. (2021) 374:abm0829. doi: 10.1126/science.abm0829 34648302 PMC9284784

[19] Liu J Chandrashekar A Sellers D Barrett J Jacob-Dolan C Lifton M . Vaccines elicit highly conserved cellular immunity to SARS-CoV-2 Omicron. Nature. (2022) 603:493–6. doi: 10.1038/s41586-022-04465-y PMC8930761 35102312

[20] Tarke A Coelho CH Zhang Z Dan JM Yu ED Methot N . SARS-CoV-2 vaccination induces immunological T cell memory able to cross-recognize variants from Alpha to Omicron. Cell. (2022) 185:847–859 e11. doi: 10.1016/j.cell.2022.01.015 35139340 PMC8784649

[21] Wagstaffe HR Thwaites RS Reynaldi A Sidhu JK McKendry R Ascough S . Mucosal and systemic immune correlates of viral control after SARS-CoV-2 infection challenge in seronegative adults. Sci Immunol. (2024) 9:eadj9285. doi: 10.1126/sciimmunol.adj9285 38335268

[22] EMA . Available online at: https://www.ema.europa.eu/en/medicines/human/EPAR/aflunov. (Accessed February 18, 2025).

[23] Galli G Medini D Borgogni E Zedda L Bardelli M Malzone C . Adjuvanted H5N1 vaccine induces early CD4+ T cell response that predicts long-term persistence of protective antibody levels. Proc Natl Acad Sci U S A. (2009) 106:3877–82. doi: 10.1073/pnas.0813390106 PMC2646626 19237568

[24] EMA . Available online at: https://www.ema.europa.eu/en/medicines/human/EPAR/pandemrix. (Accessed February 18, 2025).

[25] Weibel D Sturkenboom M Black S de Ridder M Dodd C Bonhoeffer J . Narcolepsy and adjuvanted pandemic influenza A (H1N1) 2009 vaccines - Multi-country assessment. Vaccine. (2018) 36:6202–11. doi: 10.1016/j.vaccine.2018.08.008 PMC6404226 30122647

[26] Verstraeten T Cohet C Dos Santos G Ferreira GL Bollaerts K Bauchau V . Pandemrix and narcolepsy: A critical appraisal of the observational studies. Hum Vaccin Immunother. (2016) 12:187–93. 10.1080/21645515.2015.1068486 PMC4962758 26379011

[27] EMA . Available online at: https://www.ema.europa.eu/en/documents/variation-report/pandemrix-h-c-832-ii-0079-epar-assessment-report-variation_en.pdf. (Accessed February 18, 2025).

[28] McMichael AJ Gotch FM Noble GR Beare PA. Cytotoxic T-cell immunity to influenza. N Engl J Med. (1983) 309:13–7. doi: 10.1056/NEJM198307073090103 6602294

[29] Mosmann TR McMichael AJ LeVert A McCauley JW Almond JW. Opportunities and challenges for T cell-based influenza vaccines. Nat Rev Immunol. (2024) 24:736–52. doi: 10.1038/s41577-024-01030-8 38698082

[30] Evans TG Castellino F Kowalik Dobczyk M Tucker G Walley AM Van Leuven K . Assessment of CD8(+) T-cell mediated immunity in an influenza A(H3N2) human challenge model in Belgium: a single centre, randomised, double-blind phase 2 study. Lancet Microbe. (2024) 5:645–54. doi: 10.1016/S2666-5247(24)00024-7 38729196

[31] Pedersen GK Madhun AS Breakwell L Hoschler K Sjursen H Pathirana RD . T-helper 1 cells elicited by H5N1 vaccination predict seroprotection. J Infect Dis. (2012) 206:158–66. doi: 10.1093/infdis/jis330 22551811

[32] McElhaney JE Xie D Hager WD Barry MB Wang Y Kleppinger A . T cell responses are better correlates of vaccine protection in the elderly. J Immunol. (2006) 176:6333–9. doi: 10.4049/jimmunol.176.10.6333 16670345

[33] Ciabattini A Nardini C Santoro F Garagnani P Franceschi C Medaglini D . Vaccination in the elderly: The challenge of immune changes with aging. Semin Immunol. (2018) 40:83–94. doi: 10.1016/j.smim.2018.10.010 30501873

[34] Eickhoff CS Terry FE Peng L Meza KA Sakala IG Van Aartsen D . Highly conserved influenza T cell epitopes induce broadly protective immunity. Vaccine. (2019) 37:5371–81. doi: 10.1016/j.vaccine.2019.07.033 PMC6690779 31331771

[35] Sullivan NJ Martin JE Graham BS Nabel GJ. Correlates of protective immunity for Ebola vaccines: implications for regulatory approval by the animal rule. Nat Rev Microbiol. (2009) 7:393–400. doi: 10.1038/nrmicro2129 19369954 PMC7097244

[36] Sullivan NJ Hensley L Asiedu C Geisbert TW Stanley D Johnson J . CD8+ cellular immunity mediates rAd5 vaccine protection against Ebola virus infection of nonhuman primates. Nat Med. (2011) 17:1128–31. doi: 10.1038/nm.2447 21857654

[37] Ledgerwood JE DeZure AD Stanley DA Coates EE Novik L Enama ME . Chimpanzee adenovirus vector ebola vaccine. N Engl J Med. (2017) 376:928–38. doi: 10.1056/NEJMoa1410863 25426834

[38] Stanley DA Honko AN Asiedu C Trefry JC Lau-Kilby AW Johnson JC . Chimpanzee adenovirus vaccine generates acute and durable protective immunity against ebolavirus challenge. Nat Med. (2014) 20:1126–9. doi: 10.1038/nm.3702 25194571

[39] Marzi A Engelmann F Feldmann F Haberthur K Shupert WL Brining D . Antibodies are necessary for rVSV/ZEBOV-GP-mediated protection against lethal Ebola virus challenge in nonhuman primates. Proc Natl Acad Sci U S A. (2013) 110:1893–8. doi: 10.1073/pnas.1209591110 PMC3562844 23319647

[40] Menicucci AR Sureshchandra S Marzi A Feldmann H Messaoudi I. Transcriptomic analysis reveals a previously unknown role for CD8(+) T-cells in rVSV-EBOV mediated protection. Sci Rep. (2017) 7:919. doi: 10.1038/s41598-017-01032-8 28428619 PMC5430516

[41] EMA . Available online at: https://www.ema.europa.eu/en/medicines/human/EPAR/zabdeno. (Accessed February 18, 2025).

[42] EMA . Available online at: https://www.ema.europa.eu/en/medicines/human/EPAR/mvabea. (Accessed February 18, 2025).

[43] EMA . Available online at: https://www.ema.europa.eu/en/medicines/human/EPAR/zostavax. (Accessed February 18, 2025).

[44] Sei JJ Cox KS Dubey SA Antonello JM Krah DL Casimiro DR . Effector and central memory poly-functional CD4(+) and CD8(+) T cells are boosted upon ZOSTAVAX((R)) vaccination. Front Immunol. (2015) 6:553. doi: 10.3389/fimmu.2015.00553 26579128 PMC4629102

[45] EMA . Available online at: https://www.ema.europa.eu/en/medicines/human/EPAR/shingrix. (Accessed February 18, 2025).

[46] EMA . Available online at: https://www.ema.europa.eu/en/opinion-medicine-use-outside-EU/human/dengue-tetravalent-vaccine-live-attenuated-takeda. (Accessed February 18, 2025).

[47] Marcus H Thompson E Zhou Y Bailey M Donaldson MM Stanley DA . Ebola-GP DNA prime rAd5-GP boost: influence of prime frequency and prime/boost time interval on the immune response in non-human primates. Front Immunol. (2021) 12:627688. doi: 10.3389/fimmu.2021.627688 33790899 PMC8006325

[48] Plotkin SA Correlates of protection induced by vaccination. Clin Vaccine Immunol. (2010) 17:1055–65. doi: 10.1128/CVI.00131-10 PMC2897268 20463105

[49] EMA . Available online at: https://www.ema.europa.eu/en/committees/working-parties-other-groups/emergency-task-force-etf. (Accessed February 18, 2025).

[50] EMA . Available online at: https://www.ema.europa.eu/en/qualification-novel-methodologies-medicine-development. (Accessed February 18, 2025).

[51] Prendecki M Clarke C Edwards H McIntyre S Mortimer P Gleeson S . Humoral and T-cell responses to SARS-CoV-2 vaccination in patients receiving immunosuppression. Ann Rheum Dis. (2021) 80:1322–9. doi: 10.1136/annrheumdis-2021-220626 PMC8350975 34362747

[52] Wagar LE Salahudeen A Constantz CM Wendel BS Lyons MM Mallajosyula V . Modeling human adaptive immune responses with tonsil organoids. Nat Med. (2021) 27:125–35. doi: 10.1038/s41591-020-01145-0 PMC7891554 33432170

[53] Hagan T Nakaya HI Subramaniam S Pulendran B Systems vaccinology: Enabling rational vaccine design with systems biological approaches. Vaccine. (2015) 33:5294–301. doi: 10.1016/j.vaccine.2015.03.072 PMC4581890 25858860

[54] Wang IM Bett AJ Cristescu R Loboda A ter Meulen J Transcriptional profiling of vaccine-induced immune responses in humans and non-human primates. Microb Biotechnol. (2012) 5:177–87. doi: 10.1111/j.1751-7915.2011.00317.x PMC3380551 22103427

[55] Irac SE Soon MSF Borcherding N Tuong ZK Single-cell immune repertoire analysis. Nat Methods. (2024) 21:777–92. doi: 10.1038/s41592-024-02243-4 38637691

[56] Zhang B Upadhyay R Hao Y Samanovic MI Herati RS Blair JD . Multimodal single-cell datasets characterize antigen-specific CD8(+) T cells across SARS-CoV-2 vaccination and infection. Nat Immunol. (2023) 24:1725–34. doi: 10.1038/s41590-023-01608-9 PMC10522491 37735591

